# iRNA5hmC: The First Predictor to Identify RNA 5-Hydroxymethylcytosine Modifications Using Machine Learning

**DOI:** 10.3389/fbioe.2020.00227

**Published:** 2020-03-31

**Authors:** Yuan Liu, Dasheng Chen, Ran Su, Wei Chen, Leyi Wei

**Affiliations:** ^1^College of Intelligence and Computing, Tianjin University, Tianjin, China; ^2^Center for Genomics and Computational Biology, School of Life Sciences, North China University of Science and Technology, Tangshan, China; ^3^Innovative Institute of Chinese Medicine and Pharmacy, Chengdu University of Traditional Chinese Medicine, Chengdu, China; ^4^School of Software, Shandong University, Jinan, China; ^5^Joint SDU-NTU Centre for Artificial Intelligence Research, Shandong University, Jinan, China

**Keywords:** RNA 5-hydroxymethylcytosine modification, feature representation, machine learning, web server, sequence analysis

## Abstract

RNA 5-hydroxymethylcytosine (5hmC) modification plays an important role in a series of biological processes. Characterization of its distributions in transcriptome is fundamentally important to reveal the biological functions of 5hmC. Sequencing-based technologies allow the high-throughput identification of 5hmC; however, they are labor-intensive, time-consuming, as well as expensive. Thus, there is an urgent need to develop more effective and efficient computational methods, at least complementary to the high-throughput technologies. In this study, we developed iRNA5hmC, a computational predictive protocol to identify RNA 5hmC sites using machine learning. In this predictor, we introduced a sequence-based feature algorithm consisting of two feature representations, (1) *k*-mer spectrum and (2) positional nucleotide binary vector, to capture the sequential characteristics of 5hmC sites. Afterward, we utilized a two-stage feature space optimization strategy to improve the feature representation ability, and trained a predictive model using support vector machine (SVM). Our feature analysis results showed that feature optimization can help to capture the most discriminative features. As compared to well-known existing feature descriptors, our proposed representations can more accurately separate true 5hmC from non-5hmC sites. To the best of our knowledge, iRNA5hmC is the first RNA 5hmC predictor that enables to make predictions based on RNA primary sequences only, without any need of prior experimental knowledge. Importantly, we have established an easy-to-use webserver which is currently available at http://server.malab.cn/iRNA5hmC. We expect it has potential to be a useful tool for the prediction of 5hmC sites.

## Key Points

• iRNA5hmC is the first RNA 5-hydroxymethylcytosine site predictor, which enables to make predictions based on RNA primary sequences without prior experimental knowledge.
• Benchmarking comparison results show that iRNA5hmC outperforms other machine learning algorithms trained with existing sequence-derived feature descriptors.
• Our feature analysis demonstrates that there exists the compositional and positional specificity between true 5hmC sites and non-5hmC sites.
• We have established an easy-to-use webserver that implements the predictor. It is publicly accessible at http://server.malab.cn/iRNA5hmC.

## Introduction

RNA can be decorated by various chemical modifications (Boccaletto et al., 2018). Over the past decades, more than 100 kinds of modifications have been identified in mRNA, tRNA, rRNA, and snRNA, etc. (Shi et al., 2019). These modifications play important roles in a series of biological processes (Roundtree et al., 2017), such as RNA splicing, RNA translation, and RNA decay. In addition, it was also demonstrated that RNA modifications are associated with human diseases (Jonkhout et al., 2017), including cancer, cardiovascular diseases, Bowen–Conradi syndrome, obesity, and diabetes, etc. Hence, determining their distributions in the transcriptomes is important for decoding the biological and physiological functions of RNA modifications.

Thanks to the high-throughput sequencing methods, recent years have witnessed a burst of researches on N^6^-methyladenine (m^6^A), N^1^-methyladenine (m^1^A), N^7^-methylguanosine (m^7^G), and 5-methylcytidine (m^5^C), etc. (Conde et al., 2015; Chen et al., 2019; Pian et al., 2019; Yuan et al., 2019). Another kind of RNA modification, called 5-hydroxymethylcytosine (5hmC) is formed by TET-mediated oxidation of m5C (Fu et al., 2014). The 5hmC was originally identified in wheat seedlings (Racz et al., 1978), and was also detected in various tissues of mouse and human (Li and Liu, 2011). Later on, Huber et al. (2015) found that 5hmC is pervasive in all three domains of life across a variety of different species.

Recently, by using the hMeRIP-seq method, Delatte et al. (2016) revealed a transcriptome wide profile of 5hmC in *Drosophila* and found that 5hmC modifications are non-randomly distributed, with an enrichment in coding regions. Meanwhile, they also found that 5hmC modifications are abundant in the *Drosophila* brain. A similar result was also observed by Miao et al. (2016); they found a high level of 5hmC modification enrichment in mouse brain stem, hippocampus, and cerebellum regions. These results suggest that 5hmC modification might play an important role in brain tissue. To further revealing the biological functions of 5hmC, it is necessary to characterize its distribution in the transcriptome of multiple spices. Unfortunately, the distribution of 5hmC remains uncharacterized in most species.

Considering that the high-throughput experimental methods are expensive and time-consuming, it is necessary to develop computational methods for the detection of 5hmC modification sites. Inspired by the successful application of machine learning methods for identifying RNA modifications, in this study, we developed iRNA5hmC, a computational predictor to predict RNA 5hmC sites using machine learning. In this predictor, we used the *k*-mer spectrum and positional nucleotide binary vector to respectively capture the sequence composition and position-specific characteristics of 5hmC sites, utilized a two-stage feature selection strategy to optimize the feature space, and trained the SVM-based predictive model. To the best of our knowledge, iRNA5hmC is the very first machine learning predictor that enables researchers to make RNA 5hmC predictions based on RNA primary sequences only, without any other prior experimental knowledge. Importantly, we have established an easy-to-use webserver to make the proposed predictor more impactful. We expect that it has the potential to be a complementary tool to the high-throughput sequencing methods.

## Materials and Methods

### Datasets

Here, we constructed the first 5hmC dataset for training the predictive model. It consists of positive samples and negative samples. The positive samples were collected based on Delatte et al.’s (2016) work, which contains 662 5hmC site containing sequences with the sequence similarity less than 80%. According to our previous experiences (Chen et al., 2019), the sequences were given the length of 41 nt (nucleotides) with the 5hmC site in the center. The negative samples (non-5hmC site containing sequences) were obtained by choosing 41-nt long sequences with the intermediate cytosines that are not detected as 5hmC by the hMeRIP-seq method. Accordingly, a huge number of negative samples were collected. In order to balance the number of samples between positive and negative dataset in model training, we randomly selected out 662 non-5hmC site containing sequences as the negative samples. The dataset used to train the proposed model is available at http://server.malab.cn/iRNA5hmC.

### The Proposed Predictive Framework

The predictive procedure can be concluded as two phases: (1) model training and (2) prediction. In the training phase, the training samples are encoded and integrated by feature representation algorithms. Afterward, the features are optimized to obtain the best feature subset, which are then fed into the SVM algorithm to train predictive model. In prediction phase, given the query sequences that are not characterized, we followed the similar procedure to encode the sequences, and used the trained model to predict whether or not the query sequences are 5hmC sequences. The SVM model gives each query sequence a score to measure how likely it is true 5hmC sequence. If the score is higher than 0.5, it is considered to be the 5hmC sequence; otherwise, it is not.

### Feature Representation

In this study, we introduce a feature representation algorithm containing the following two sequence-based feature descriptors: (1) *k*-mer spectrum and (2) nucleotide binary encoding, which are described as follows.

The first feature descriptor is *k*-mer spectrum. There are two reasons for using it. One is that it is a simple and useful feature algorithm to encode character sequences like RNAs and DNAs. On the other hand, more importantly, previous study has demonstrated that DNA 5mC is often found in contexts of CG, CHG, and CHH (H represents either A, C, or T) (Kumar et al., 2018). Therefore, there might be similar for RNA 5hmC modification.

For convenience of discussions, a given RNA sequence can be represented as

(1) $$S=R_{1}\,R_{2}\,\cdots \,R_{i}\,\cdots \,R_{L-1}\,R_{L}$$

where *R*_1_ represents the first nucleotide, *R*_2_ represents the second nucleotide, and so forth. *R*_*i*_ can be any of the four nucleotides {A, C, U, G}. The *k*-mer spectrum computes the occurrence frequencies of all possible sequential patterns with length *k*. Therefore, using this descriptor, the given sequence can be represented as,

(2) $$F^{k\,\text{-mer}}=\left[f_{1}^{k\,\text{-mer}},f_{2}^{k\,\text{-mer}},\ldots ,f_{i}^{k\,\text{-mer}},\ldots ,f_{4^{k}}^{k\,\text{-mer}}\right]$$

where $f_{i}^{k\,\text{-mer}}$ is the occurrence frequency of the *i-*th *k*-mer in *S*. Similarly, we used 2-mer and 3-mer spectrum to encode our RNA sequences. Naturally, *S* is represented as 2-mer and 3-mer vector, respectively:

(3) $$F^{2-\mathrm{mer}}=\left[f\,\left(A\,A\right),f\,\left(A\,C\right),\ldots ,f\,\left(G\,G\right)\right]$$

(4) $$F^{3-\mathrm{mer}}=\left[f\,\left(A\,A\,A\right),f\,\left(A\,A\,C\right),\ldots ,f\,\left(G\,G\,G\right)\right]$$

The second feature descriptor is nucleotide binary encoding, in which we transform different nucleotides into different numeric vectors by the following rule: the codes of “A,” “U,” “C,” and “G” are “0001,” “0010,” “0100,” and “1000,” respectively.

Finally, a given RNA sequence is encoded as a total of 244 features (41×4 + 4^2^ + 4^3^ = 244).

### Feature Optimization

Feature optimization is a key step to remove the noisy features and retain the features having the highest degree of separability between two classes, which has been employed to improve the predictive performance in several bioinformatics problems. In this study, we used a two-stage feature selection strategy. In the first step, we compute the feature importance for the 244 features by analysis of variance (ANOVA) (Chen et al., 2016), which calculates the separability degree of each feature to obtain respective *F*-value and yields a feature ranking list regarding their classification importance. The feature with a larger *F*-value indicates much more importance. The ANOVA *F*-value of the θ-th feature definitions is given below:

(5) $$F-\text{value}\,\left(\theta \right)=\frac{S_{B\,\left(\theta \right)}^{2}}{S_{w\,\left(\theta \right)}^{2}}$$

where $S_{B\,\left(\theta \right)}^{2}$ and $S_{w\,\left(\theta \right)}^{2}$ are the means square between (MSB) and means square within (MSW), respectively. They are defined as follows:

(6) $$S_{B\,\left(\theta \right)}^{2}=\frac{1}{d\,f_{B}}\,\sum_{i=1}^{K}n_{i}\,{\left(\frac{\sum_{j=1}^{n_{i}}f_{i\,j}\,\left(\theta \right)}{n_{i}}-\frac{\sum_{i=1}^{K}\sum_{j=1}^{n_{i}}f_{i\,j}\,\left(\theta \right)}{\sum_{i=1}^{K}n_{i}}\right)}^{2}$$

(7) $$S_{w}^{2}\,\left(\theta \right)=\frac{1}{d\,f_{w}}\,\sum_{i=1}^{K}\sum_{j=1}^{n_{i}}{\left(f_{i\,j}\,\left(\theta \right)-\frac{\sum_{i=1}^{K}\sum_{j=1}^{n_{i}}f_{i\,j}\,\left(\theta \right)}{\sum_{i=1}^{K}n_{i}}\right)}^{2}$$

here *df*_*B*_ = *K*−1 and *df*_*w*_ = *N*−*K* are degrees of freedom for MSB and MSW, respectively. *K* and *N* represent the number of groups (for the current case *K* = 2) and total number of samples, respectively; and *n*_*i*_ is the number of sample in the *i*-th group. *f*_*ij*_(θ) denotes feature value of the θ-th feature of the *j*-th sample in the *i-*th group.

In the second step, we used the sequential forward search (SFS) strategy to determine the optimal feature representations (Whitney, 2006). To be specific, features from the ranked feature list are added ten-by-ten from lower rank (higher index) to higher rank (lower index) each time, and are used to re-construct the SVM-based prediction model on the five-fold cross validation test. Finally, the feature subset with the best performance (in terms of ACC) is recognized as the optimal set. The detail of the feature optimization results is discussed in section “Feature analysis.”

### Classification Algorithm

Support vector machine is a powerful machine learning algorithm for classification, regression as well as other machine learning tasks. It has been successfully applied to a series of supervised learning problems in computational biology (Bu et al., 2018; Zhang et al., 2018; Li and Liu, 2019; Liu and Li, 2019; Liu et al., 2019). The main principle of SVM is to transform the input data into high-dimensional feature space, and then determine the most suitable hyperplane for separating the samples in one class from another. After that, the hyperplane can be used to predict the class of unknown data. In this study, we implemented the SVM algorithm by using the SVM library in Python (version 2.7.15). We chose the radial basis function (RBF) as the kernel function, which can transform the non-linearly separated feature space into higher-dimensional one that is linearly separable. Moreover, we optimized the parameters by grid search to determine the optimal classification hyperplane for SVM algorithm. The classification algorithm optimization results can be seen in section “Classifier Optimization.”

### Evaluation Metrics and Methods

Four metrics, namely sensitivity (Sn), specificity (Sp), accuracy (ACC) and Matthew’s correlation coefficient (MCC), were used to quantitatively evaluate the performance of the proposed method. Their definitions are given below:

(8) $$\left\{ \begin{array}{c} \text{SN}=\frac{\text{TP}}{\text{TP}+\text{FN}}\\ \text{SP}=\frac{\text{TN}}{\text{TN}+\text{FP}}\\ \text{ACC}=\frac{\text{TP}+\text{TN}}{\text{TP}+\text{TN}+\text{FN}+\text{FP}}\\ \text{MCC}=\frac{\text{TP}\times \text{TN}-\text{FP}\times \text{FN}}{\sqrt{\left(\text{TP}+\text{FN}\right)\,\left(\text{TP}+\text{FP}\right)\,\left(\text{TN}+\text{FP}\right)\,\left(\text{TN}+\text{FN}\right)}} \end{array}\right.$$

where TP (true positive) represents the number of correctly predicted positive samples; TN (true negative) represents the number of correctly predicted negative samples; FP (false positive) represents the number of negative samples incorrectly predicted to positive samples; FN (false negative) represents the number of positive samples incorrectly predicted to negative samples.

Moreover, we used the five-fold cross validation method to measure the predictive performance of the predictor (Liu, 2019). The procedure of this validation method involves three steps. Firstly, a dataset is randomly partitioned into five equal-size subsets. Of the five subsets, four are chosen as the training dataset for model training, while the remaining one is retained as the validation data to evaluate the performance of the model. After that, this process is repeated until each subset is used exactly once as the validation data. Lastly, the five results are averaged to obtain a final prediction estimation.

To more intuitively evaluate the predictive performance, we also used two curves: receiver operating characteristic (ROC) curve and Precision-Recall (PR) curve. The ROC curve plots the true positive rate (TPR) against the false positive rate (FPR; 1-specificity) under different classification thresholds; while the PR curve plots precision (the fraction of TP in all predicted positives) against recall (sensitivity) at various threshold settings. The PR curve is more sensitive to false positives than the ROC curve, especially evaluated on imbalanced dataset. In addition, the area under the ROC curve (AUC) is utilized to quantitatively measure the quality of the predictive model. The range of AUC is 0.5–1. The higher the AUC is, the better the predictor (Hanley and McNeil, 1982).

## Results and Discussion

### Classifier Optimization

To achieve the best performance, we conducted the following experiments to optimize the SVM classifier.

**FIGURE 1:**
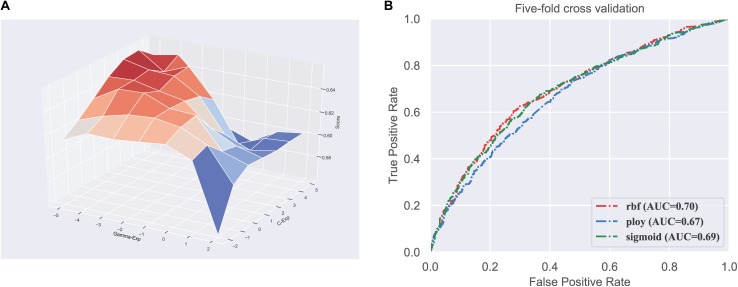
Parameter and kernel optimization of the SVM. **(A)** Visualization of classifier parameter optimization based on grid search; **(B)** ROC curves of different kernels in SVM.

Firstly, we did the parameter optimization. There are two parameters in SVM, including the penalty coefficient (denoted as *c*) and gamma (denoted as *g*). We used the grid search strategy to find the optimal values of log2c and log2g in the range (−2 to 5) and (−5 to 2), respectively. Figure 1A shows the visualization of the grid search process in three-dimensional space.

Next, we need to determine which kernel function is most suitable for our dataset. There are three kernel functions in SVM, including RBF, Polynomial, and Sigmoid, for handling different feature space. Therefore, we compared the performance of the three kernels. We can observe in Figure 1B that the RBF performs better than the other two kernels, with the highest AUC of 0.70. Consequently, the SVM with RBF kernel is used to train the model in our predictor.

### Feature Analysis

To in-depth explore the critical information benefiting for the prediction of 5hmC, we conducted a series of feature analysis experiments, including feature combination, optimization, and contribution analysis.

#### Feature Combination Analysis

In our predictive framework, three feature descriptors, including 2-mer spectrum, 3-mer spectrum, and nucleotide binary features are concatenated to encode RNA sequences. To evaluate their contributions for 5hmC prediction, we compared the performance of different features and that of their combinations. The results are listed in Table 1. As can be seen, amongst the three individual feature descriptors, the 3-mer spectrum performs the best than the other two (2-mer spectrum and binary vector). This indicates that the sequential patterns are more useful for 5hmC prediction. By combining 2-mer and 3-mer spectrum, the performance is slightly improved. Particularly, adding binary vector to the combination of 2-mer and 3-mer spectrum, the performance decreases dramatically to 56.1% and 0.122 in terms of ACC and MCC, respectively, which is almost the same with the performance by using binary vector only. The possible reason is that integrating different types of feature space results in mutual information that is not useful for the performance.

**TABLE 1:** Five-fold cross validation results of different features and their combinations.

**Features**	**ACC (%)**	**SN (%)**	**SP (%)**	**MCC**
A	62.3	61.8	62.8	0.246
B	64.0	63.4	64.5	0.279
C	53.3	53.5	53.2	0.066
A + B	64.0	62.7	65.3	0.280
A + C	55.4	55.9	54.8	0.107
B + C	55.8	57.1	54.5	0.116
A + B + C	56.1	57.6	54.7	0.122

*A, 2-mer spectrum; B, 3-mer spectrum; C, binary vector; +, operation of combining.*

#### Feature Optimization Analysis

**FIGURE 2:**
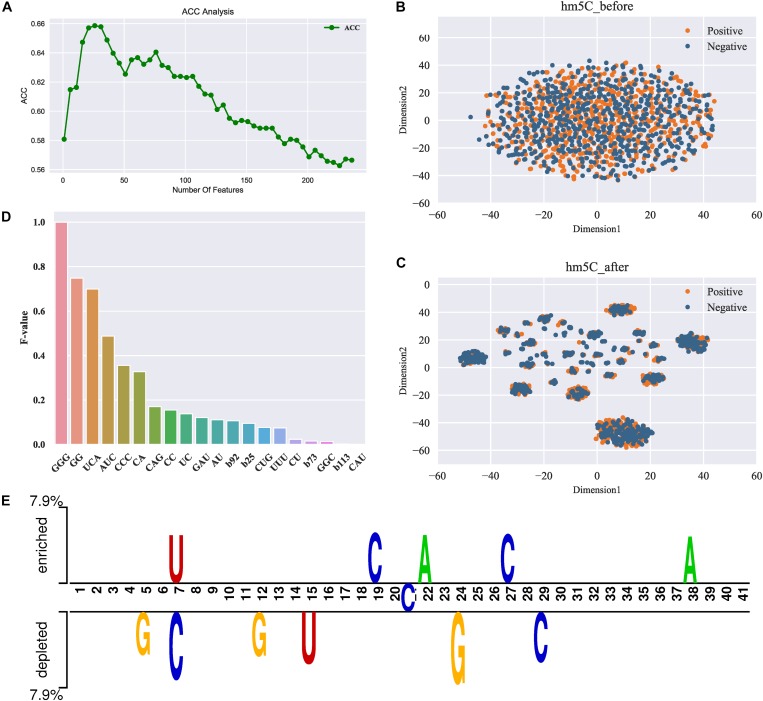
Feature analysis results. **(A)** ACC curve of the feature selection; **(B,C)** represent the distribution visualization of the samples (positive and negative) in feature space before and after feature optimization, respectively; **(D)** *F*-values of the top 20 most important features. Note that the *x*-axis represents the specific features and the *y*-axis represents the *F*-value. Note that b92 denotes the 92th feature of the binary vector, b25 denotes the 25th feature, and so forth; **(E)** TSL (Two Sample Logos) visualization of the positives and negatives in the dataset used in this study.

To obtain the most discriminative features, we further did the two-stage feature optimization to the integrated feature space. The procedure of the optimization strategy can be seen in section “Methods and Materials.” Figure 2A illustrates the ACC curve of the predictive model by gradually adding features (from the feature rank list) under the SFS process. As shown in Figure 2A, when the feature number reaches to 26, the model achieves the maximum ACC. After reaching the peak, the performance leads to a significant drop as adding more features (see Figure 2A). This suggests most of the low-ranked features (binary vector) are relatively irrelevant with the high-ranked features, and even result in a decrease in the performance. The significant improvement by the optimal features is observed, for which the overall performances in terms of ACC and MCC were increased approximately 9.38% and 0.188 after feature optimization. These results demonstrate that feature optimization can effectively enhance the feature representation ability, thereby contributing to the improved performance.

Next, we further compared the spatial distribution of the original feature space and the optimal feature space. For intuitive comparison, we used a visualization tool t-SNE (Maaten and Hinton, 2008) that enable to reduce the feature space to a two-dimensional space. Figures 2B,C depict the t-SNE visualization of the original and optimal feature space, respectively. As can be seen from Figure 2B, the positive (true 5hmC sites) and negative (non-5hmC sites) samples in the original feature space are mixed up, indicating that the original feature space cannot separate true 5hmC sites from non-5hmC sites well. In contrast, after feature optimization (see Figure 2C), the positives and negative samples in feature space are distributed in relatively clear clusters. This demonstrates that feature optimization is able to remove some irrelevant features and learn the most representatives of true 5hmC sites.

#### Feature Contribution Analysis

To specify which features are important for the prediction of 5hmC, we further analyzed the importance of different features in our feature set. The details regarding how to calculate the feature importance can be referred to section “Feature Optimization.” Figure 2D illustrates the importance scores (*F*-value) of the top 20 features, and the detail of all the features can be found in Supplementary Material. As shown in Figure 2D, amongst the top 20 features, most of the features are *k-*mer spectrum (3-mer and 2-mer) while only 4 of the 20 are binary features, indicating that there exist significant compositional differences between the positive and negative samples. In particular, the sequential patterns “GGG” and “GG” are the most important features, indicating that the compositions of the *guanine* (G) nucleotide are discriminative features for the prediction of 5hmC. This observation is different from the fact that DNA 5mC is often found in contexts of CG or C × G (Kumar et al., 2018). We further used Two Sample Logos (TSL) (Vacic et al., 2006), a web-based application to calculate and visualize differences between two sets (the positive and negative) of aligned samples of nucleotides. Figure 2E depicts the TSL visualization of the positive and negative samples in our dataset. We observed that the enrichment of nucleotides is significantly different in specific positions along the sequences between the positive and negative samples. For example, the *adenine* (A) nucleotide is enriched at 38th position in the positive set while not in the negative set. This demonstrates that the compositional features might have the positional preference. Therefore, exploring positional features is probably helpful for the further performance improvement.

### Comparison of Our Feature Set With Existing Feature Algorithms

In this section, we compared the proposed features and four sequence-based feature descriptors, including PCP (physical–chemical properties), MMI (multivariate mutual information), PseDNC (pseudo dinucleotide composition), and PseEIIP (electron-ion interaction pseudopotentials of trinucleotide). The compared feature descriptors explore sequential information from different aspects. For example, PCP uses the physical–chemical properties of dinucleotides and explores the correlation between any two nucleotides using auto-covariance and cross covariance transformations (Liu et al., 2015; Wei et al., 2019). MMI calculates the multivariate mutual information of nucleotides (Wei et al., 2019). Pse-DNC can capture the local and global characteristic patterns by integrating the sequence-order information with PCP (Chen et al., 2014). More details of the feature descriptors can be referred to (Wei et al., 2019). We evaluated all the feature descriptors including our feature set on the same data set with five-fold cross validation. Since our feature set is optimized using the feature optimization strategy, for the purpose of fair comparison, we also used the same strategy to optimize the four compared feature descriptors. The obtained results by using different features were reported in Table 2.

**TABLE 2:** Five-fold cross validation results of the proposed feature set with other sequence-based feature descriptors.

**Features**	**ACC (%)**	**SN (%)**	**SP (%)**	**MCC**
PCP	63.97	68.73	59.21	0.2807
MMI	61.56	63.14	59.97	0.2312
PseDNC	62.84	61.33	64.35	0.2569
PseEIIP	64.27	69.64	58.91	0.2872
Our feature set	**65.48**	67.67	63.29	**0.3100**

*The bold value indicates the highest value of this column.*

As seen in Table 2, our feature set performs better than other sequence-based feature descriptors in terms of ACC and MCC, with exceptions of SN and SP. The ACC and MCC of our feature set is 65.48% and 0.31, respectively, which are 1.2% and 0.023 higher than that of the runner-up feature descriptor – PseEIIP, with the ACC of 64.27% and MCC of 0.2872. It is worth noting that our SN and SP are 67.67% and 63.29%, slightly worse than the best descriptor – PseEIIP in SN and PseDNC in SP, respectively. Although our SN and SP are not the best, they are more balanced as compared to PseEIIP and PseDNC, thus contributing to the highest overall performance. This indicates that our feature set is more effective to distinguish true 5hmC sites from non-5hmC sites. In addition, since the majority of our feature set is *k*-mer spectrum features, this also demonstrates that the sequential patterns is capable of better capturing the characteristics of 5hmC sites as compared to other information like PCP and nucleotide mutual information, and so on.

### Comparison With Different Classification Algorithms

**FIGURE 3:**
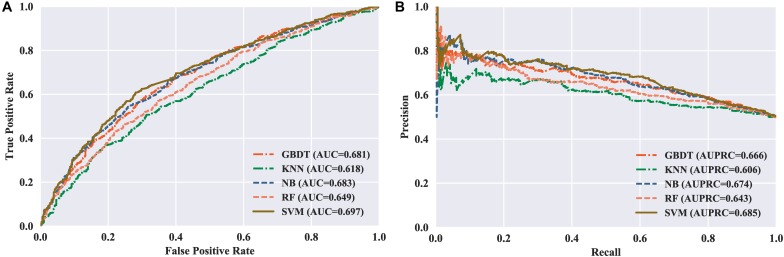
Performance of different classifiers evaluated with five-fold cross validation. **(A)** ROC curves of different classifiers. **(B)** PR curves of different classifiers.

To measure the effectiveness of SVM, we compared its performance with multiple well-known classifiers, like gradient boosting decision tree (GBDT) (Liao et al., 2018), k-nearest neighbor (KNN), logistic regression (LR), naive Bayes (NB) (Feng et al., 2013), and random forest (RF) (Lv et al., 2019; Ru et al., 2019; Wei et al., 2017). For fair comparison, we trained the classifiers on the same dataset with our feature set, and then fine-tuned the classifiers one by one to achieve the optimal performance. The models are also evaluated by five-fold cross validation, and the evaluation results are presented in Table 3. We can see that the SVM achieves ACC of 65.48%, SN of 67.67%, SP of 63.29%, and MCC of 0.31, respectively, outperforming the other four classifiers in two out of the four metrics: MCC and ACC. To be specific, our ACC and MCC are higher than that of the runner-up GBDT by 1.88% and 0.0381, respectively. Additionally, we further intuitively compared the performance of different classifiers using ROC and PR curves as shown in Figures 3A,B, respectively. The results demonstrate that the SVM classifier has the better discriminative power to distinguish the 5hmC sites from non-5hmC sites than the other four classifiers in this study.

**TABLE 3:** Comparative results of SVM and four well-known classifiers on the dataset used in this study.

**Classifiers**	**ACC (%)**	**SN (%)**	**SP (%)**	**MCC**
GBDT	63.60	63.90	63.29	0.2719
KNN	58.46	56.95	59.97	0.1693
NB	63.37	63.00	63.75	0.2674
RF	60.27	62.08	58.46	0.2056
SVM (this study)	**65.48**	67.67	63.29	**0.3100**

*The bold value indicates the highest value of this column.*

### Webserver Implementation

For the convenience of researchers, we established an easy-to-use webserver that implements our predictor, which is freely available at http://server.malab.cn/iRNA5hmC. Below, we give researchers a step-by-step guideline on how to use the webserver to get the desired prediction results. Firstly, users need to submit their query RNA sequences into the input box. Note that the input sequences should be in FASTA format. After that, users can specify the prediction confidence from 0 to 1. Otherwise, under default setting, the query sequence is predicted as true 5hmC sequence if the prediction confidence is >0.5. Afterward, clicking on the “Submit” button, users can obtain the desired results on the screen of the computer.

## Conclusion

In this study, we have proposed a computational predictor namely iRNA5hmC to predict RNA 5hmC sites using machine learning. To the best of our knowledge, this is the first RNA 5hmC predictor that enables to make predictions based on RNA primary sequences only, without any other prior experimental knowledge. In particular, we have established an easy-to-use webserver for researchers to make the proposed predictor more impactful and have the potential to be complementary tool to the high-throughput sequencing methods. However, we have to see there still has some aspects, such as the relatively low predictive performance, and small-size dataset, which need to be improved in our future work.

## Data Availability Statement

The datasets generated for this study can be found in the http://server.malab.cn/iRNA5hmC.

## Author Contributions

LW, WC, and RS conceived and designed the experiments. WC acquired the experiment data. DC and YL performed the study. DC and RS carried out the data analysis. YL, RS, and LW wrote the manuscript. All authors read and approved the final manuscript.

## Conflict of Interest

The authors declare that the research was conducted in the absence of any commercial or financial relationships that could be construed as a potential conflict of interest.
