# Supplementary material for: iRNA5hmC: The First Predictor to Identify RNA 5-Hydroxymethylcytosine Modifications Using Machine Learning
Source: Front Bioeng Biotechnol. 2020 Mar 31;8:227. doi: 10.3389/fbioe.2020.00227 (PMC7137033; doi:10.3389/fbioe.2020.00227)
Supplement: Supplementary file 1 [file Table_1.docx]

**Supplementary tables**

**Table S1.** **The importance scores (F-value) of the features**

| Rank | Feature | F-value | Rank | Feature | F-value | Rank | Feature | F-value |
| --- | --- | --- | --- | --- | --- | --- | --- | --- |
| 1 | GGG | 1 | 41 | AG | 0.065374 | 81 | b15 | 0.029091 |
| 2 | GG | 0.775506 | 42 | b119 | 0.062785 | 82 | b114 | 0.028823 |
| 3 | UCA | 0.731451 | 43 | b95 | 0.061483 | 83 | UUC | 0.027884 |
| 4 | AUC | 0.543728 | 44 | b104 | 0.058444 | 84 | AGA | 0.027689 |
| 5 | CCC | 0.425752 | 45 | b86 | 0.057474 | 85 | b68 | 0.026933 |
| 6 | CA | 0.400636 | 46 | b140 | 0.056966 | 86 | CUA | 0.025745 |
| 7 | CAG | 0.260484 | 47 | b40 | 0.054478 | 87 | b161 | 0.025509 |
| 8 | CC | 0.246664 | 48 | b156 | 0.053454 | 88 | b103 | 0.025327 |
| 9 | UC | 0.231935 | 49 | UU | 0.05335 | 89 | b72 | 0.02499 |
| 10 | GAU | 0.216643 | 50 | b59 | 0.053008 | 90 | UUA | 0.024932 |
| 11 | AU | 0.207643 | 51 | UAG | 0.050256 | 91 | CGU | 0.023969 |
| 12 | b92 | 0.203664 | 52 | b148 | 0.04914 | 92 | b99 | 0.023809 |
| 13 | b25 | 0.19333 | 53 | UAC | 0.049007 | 93 | UCU | 0.023756 |
| 14 | CUG | 0.177272 | 54 | AUA | 0.0481 | 94 | b88 | 0.02325 |
| 15 | UUU | 0.1753 | 55 | UGC | 0.04786 | 95 | b84 | 0.022992 |
| 16 | CU | 0.129563 | 56 | GAC | 0.046164 | 96 | CAA | 0.022441 |
| 17 | b73 | 0.122064 | 57 | b149 | 0.045982 | 97 | b32 | 0.022035 |
| 18 | GGC | 0.120925 | 58 | GAG | 0.044755 | 98 | b61 | 0.021512 |
| 19 | b113 | 0.110969 | 59 | b128 | 0.043664 | 99 | b62 | 0.020941 |
| 20 | CAU | 0.108721 | 60 | GCU | 0.0428 | 100 | b129 | 0.020517 |
| 21 | b105 | 0.106958 | 61 | b3 | 0.041504 | 101 | b123 | 0.019174 |
| 22 | GUG | 0.103017 | 62 | b115 | 0.038922 | 102 | b155 | 0.019115 |
| 23 | AC | 0.100487 | 63 | b18 | 0.038922 | 103 | b150 | 0.017955 |
| 24 | b58 | 0.0988 | 64 | b0 | 0.038671 | 104 | b46 | 0.017825 |
| 25 | CCG | 0.097477 | 65 | ACA | 0.03759 | 105 | b48 | 0.017553 |
| 26 | AGC | 0.094977 | 66 | b116 | 0.036679 | 106 | b89 | 0.01714 |
| 27 | UGG | 0.094823 | 67 | b57 | 0.03658 | 107 | b77 | 0.016842 |
| 28 | CGG | 0.092661 | 68 | b108 | 0.036046 | 108 | b121 | 0.016651 |
| 29 | b26 | 0.090864 | 69 | GUU | 0.035829 | 109 | GGA | 0.016461 |
| 30 | UA | 0.086876 | 70 | b78 | 0.035595 | 110 | UUG | 0.015969 |
| 31 | GCA | 0.086474 | 71 | b158 | 0.035567 | 111 | AUG | 0.015737 |
| 32 | b151 | 0.080996 | 72 | CAC | 0.0348 | 112 | b79 | 0.015264 |
| 33 | b44 | 0.080742 | 73 | b154 | 0.03435 | 113 | b42 | 0.014775 |
| 34 | b87 | 0.079792 | 74 | GGU | 0.033609 | 114 | b147 | 0.014658 |
| 35 | b16 | 0.075749 | 75 | b11 | 0.032079 | 115 | b69 | 0.014562 |
| 36 | GCG | 0.073973 | 76 | UAA | 0.031883 | 116 | b75 | 0.014492 |
| 37 | ACG | 0.070169 | 77 | b102 | 0.031474 | 117 | UCG | 0.014388 |
| 38 | b163 | 0.069282 | 78 | b27 | 0.031328 | 118 | b47 | 0.013795 |
| 39 | ACU | 0.06909 | 79 | b33 | 0.030579 | 119 | b37 | 0.013785 |
| 40 | AAA | 0.067155 | 80 | b4 | 0.02991 | 120 | b50 | 0.013501 |

| Rank | Feature | F-value | Rank | Feature | F-value | Rank | Feature | F-value |
| --- | --- | --- | --- | --- | --- | --- | --- | --- |
| 121 | b143 | 0.013103 | 161 | ACC | 0.006461 | 201 | b30 | 0.001152 |
| 122 | b8 | 0.013059 | 162 | b144 | 0.005987 | 202 | b14 | 0.001121 |
| 123 | b153 | 0.012999 | 163 | b90 | 0.005797 | 203 | b74 | 0.00109 |
| 124 | AA | 0.012867 | 164 | b6 | 0.005549 | 204 | AUU | 0.001081 |
| 125 | AAU | 0.012555 | 165 | b39 | 0.005493 | 205 | b124 | 0.001079 |
| 126 | b117 | 0.012547 | 166 | b7 | 0.005439 | 206 | AGG | 0.000792 |
| 127 | b120 | 0.012242 | 167 | b67 | 0.005245 | 207 | b157 | 0.000762 |
| 128 | b96 | 0.012193 | 168 | b152 | 0.0051 | 208 | b9 | 0.000738 |
| 129 | b160 | 0.012145 | 169 | b12 | 0.004887 | 209 | b45 | 0.000702 |
| 130 | b2 | 0.012145 | 170 | b24 | 0.004844 | 210 | b136 | 0.00068 |
| 131 | b142 | 0.01196 | 171 | b133 | 0.004822 | 211 | b34 | 0.000644 |
| 132 | GCC | 0.011884 | 172 | GUA | 0.004623 | 212 | b127 | 0.000621 |
| 133 | b134 | 0.011786 | 173 | b66 | 0.004362 | 213 | b70 | 0.000612 |
| 134 | GAA | 0.011752 | 174 | AAG | 0.00408 | 214 | b71 | 0.000602 |
| 135 | b106 | 0.011743 | 175 | b29 | 0.00382 | 215 | b101 | 0.000388 |
| 136 | b139 | 0.011425 | 176 | b112 | 0.003562 | 216 | b5 | 0.000338 |
| 137 | CG | 0.011334 | 177 | b64 | 0.00352 | 217 | b41 | 0.000336 |
| 138 | b60 | 0.010493 | 178 | b76 | 0.003334 | 218 | b137 | 0.000322 |
| 139 | b53 | 0.010306 | 179 | b97 | 0.003328 | 219 | b36 | 0.000316 |
| 140 | b23 | 0.01006 | 180 | AGU | 0.003104 | 220 | b28 | 0.000294 |
| 141 | b1 | 0.010025 | 181 | b17 | 0.003023 | 221 | b19 | 0.000276 |
| 142 | b138 | 0.010023 | 182 | GC | 0.002797 | 222 | b162 | 0.000274 |
| 143 | b93 | 0.009972 | 183 | b126 | 0.002701 | 223 | b131 | 0.000272 |
| 144 | b10 | 0.009952 | 184 | b52 | 0.002701 | 224 | b38 | 0.000266 |
| 145 | b65 | 0.00977 | 185 | CGA | 0.002693 | 225 | GU | 0.000223 |
| 146 | GA | 0.009571 | 186 | b146 | 0.002633 | 226 | b21 | 7.83E-05 |
| 147 | b43 | 0.009535 | 187 | b130 | 0.002496 | 227 | UGU | 7.27E-05 |
| 148 | CUC | 0.009489 | 188 | b35 | 0.002445 | 228 | b100 | 7.10E-05 |
| 149 | b56 | 0.00918 | 189 | b159 | 0.002369 | 229 | AAC | 6.95E-05 |
| 150 | b109 | 0.008639 | 190 | GUC | 0.002368 | 230 | b31 | 6.85E-05 |
| 151 | b20 | 0.008595 | 191 | CGC | 0.002151 | 231 | b111 | 6.51E-05 |
| 152 | b110 | 0.008499 | 192 | b125 | 0.00206 | 232 | UAU | 1.68E-05 |
| 153 | b94 | 0.00829 | 193 | b132 | 0.00194 | 233 | CCA | 1.59E-05 |
| 154 | b63 | 0.007927 | 194 | b118 | 0.001796 | 234 | UG | 5.32E-06 |
| 155 | CCU | 0.007728 | 195 | b107 | 0.001689 | 235 | b141 | 0 |
| 156 | b85 | 0.007637 | 196 | b55 | 0.001684 | 236 | b54 | 0 |
| 157 | UGA | 0.007276 | 197 | b91 | 0.001637 | 237 | b22 | 0 |
| 158 | b122 | 0.007116 | 198 | b51 | 0.001637 | 238 | b145 | 0 |
| 159 | UCC | 0.007047 | 199 | CUU | 0.001415 | 239 | b98 | 0 |
| 160 | b13 | 0.006639 | 200 | b49 | 0.001329 | 240 | b135 | 0 |
